# Impact of including productivity costs in economic analyses of vaccines for C. difficile infections and infant respiratory syncytial virus, in a UK setting

**DOI:** 10.1186/s12962-024-00533-4

**Published:** 2024-04-30

**Authors:** Margherita Neri, Janne C. Mewes, Fernando Albuquerque de Almeida, Sophia Stoychev, Nadia Minarovic, Apostolos Charos, Kimberly M. Shea, Lotte M.G. Steuten

**Affiliations:** 1 Office of Health Economics (OHE), London, UK; 2 Panaxea b.v, Amsterdam, The Netherlands; 3 Pfizer Inc, New York, USA

**Keywords:** Respiratory syncytial virus, C. difficile infection, Vaccine, Productivity losses, Economic evaluation

## Abstract

**Objectives:**

It has been estimated that vaccines can accrue a relatively large part of their value from patient and carer productivity. Yet, productivity value is not commonly or consistently considered in health economic evaluations of vaccines in several high-income countries. To contribute to a better understanding of the potential impact of including productivity value on the expected cost-effectiveness of vaccination, we illustrate the extent to which the incremental costs would change with and without productivity value incorporated.

**Methods:**

For two vaccines currently under development, one against Cloistridioides difficile (C. difficile) infection and one against respiratory syncytial disease (RSV), we estimated their incremental costs with and without productivity value included and compared the results.

**Results:**

In this analysis, reflecting a UK context, a C. difficile vaccination programme would prevent £12.3 in productivity costs for every person vaccinated. An RSV vaccination programme would prevent £49 in productivity costs for every vaccinated person.

**Conclusions:**

Considering productivity costs in future cost-effectiveness analyses of vaccines for C. difficile and RSV will contribute to better-informed reimbursement decisions from a societal perspective.

**Supplementary Information:**

The online version contains supplementary material available at 10.1186/s12962-024-00533-4.

## Introduction

Productivity costs occur when illness, disability, or premature death cause a production loss. Such losses can occur in both paid and unpaid work, among individuals affected by disease and their informal caregivers. The COVID-19 pandemic has shown the tremendous impact that uncontrolled spread of infectious diseases can have on productivity costs, as well as the potential for vaccines to reduce this impact [1].

While a similar magnitude of impact may not be expected from every vaccine, productivity value is by no means a unique value element of vaccines against SARS-Cov-2 viruses. In fact, productivity value has long been reported as a significant component of the value of vaccines [2–6]. Yet, the value generated by vaccines in preventing productivity costs is not commonly or consistently considered in economic evaluations of vaccines in several high-income countries [7] especially those that take a healthcare systems perspective to inform coverage and reimbursement decisions, e.g. the Joint Committee on Vaccination and Immunisation in England which largely follows the methods set out by the National Institute of Health and Care Excellence [8]. Other reasons for not considering productivity costs include equity concerns, controversies around the appropriate calculation method, and data limitations, which we have previously described elsewhere [9]. Excluding productivity costs, however, risks undervaluation of immunisation programmes, which in the long-term may have negative consequences on research and development incentives for vaccines and detrimental effects on population health and a country’s economic performance (10–11).

This study, therefore, aims to illustrate to what extent the inclusion of productivity costs might impact the value assessment of vaccines against Cloistridioides difficile infections (C. difficile) and infant respiratory syncytial virus (RSV) infections, which are currently in development or have recently been launched [12, 13]. C. difficile is a bacterium that can infect the bowel and cause diarrhoea. In England, around 12,500 C. difficile cases occurred in the financial year 2020/2021 [14]. RSV is a seasonal disease that affects approximately 33.8 million children under the age of five worldwide per year [15].

While health economic evaluations of preventive interventions and potential vaccine candidates for C. difficile and RSV have mostly considered their healthcare costs, there may be substantial productivity costs involved in each though the types of productivity losses incurred were expected to differ substantially between the two diseases. C. difficile can cause losses in productivity among patients in working age, their informal caregivers, and losses from unpaid voluntary work [16]. Productivity losses for RSV are mainly incurred by carers of children, e.g. when taking time off to accompany their child to GP appointments, or when the child is too sick to attend day care. Moreover, RSV-mortality incurs productivity losses in the form of lifetime lost income for the child.

This paper approximates the *incremental costs* of no vaccination over vaccination, *with and without consideration of productivity costs* in a UK setting. As such, the sole focus of this research lies on the potential impact of including versus excluding productivity costs, and a de-novo analysis of the full societal cost-effectiveness of C. difficile and RSV vaccination programmes was beyond the scope of this study.

## Methods

We developed disease-specific models to estimate the expected direct healthcare costs and productivity costs under the standard of care (i.e. no vaccination programme) and in the presence of a vaccination programme. The main outcome of interest was the difference in incremental costs with and without productivity costs.

To estimate direct healthcare costs and productivity costs, we used published cost-effectiveness studies of C. difficile and RSV preventative interventions relevant to UK setting [17, 18]. We included productivity costs due to losses in paid work incurred by patients and caregivers in working age, and due to informal care provided by caregivers in non-working age. We excluded the cost of the vaccine from the analyses to strictly compare the difference in all other cost components, net of any recoup of value by a vaccine manufacturer. The model structure and elements of productivity cost for each disease are detailed in the Sect. 2.1 and 2.2.

Production losses in paid work were valued according to the human capital approach [19]. Productivity costs associated to informal care were estimated using the opportunity cost approach, which values the benefits forgone in informal caregiving time at the market´s gross wage rate [20]. The model input data are described in Sect. 2.3.

### C. Difficile model characteristics

**Fig. 1:**
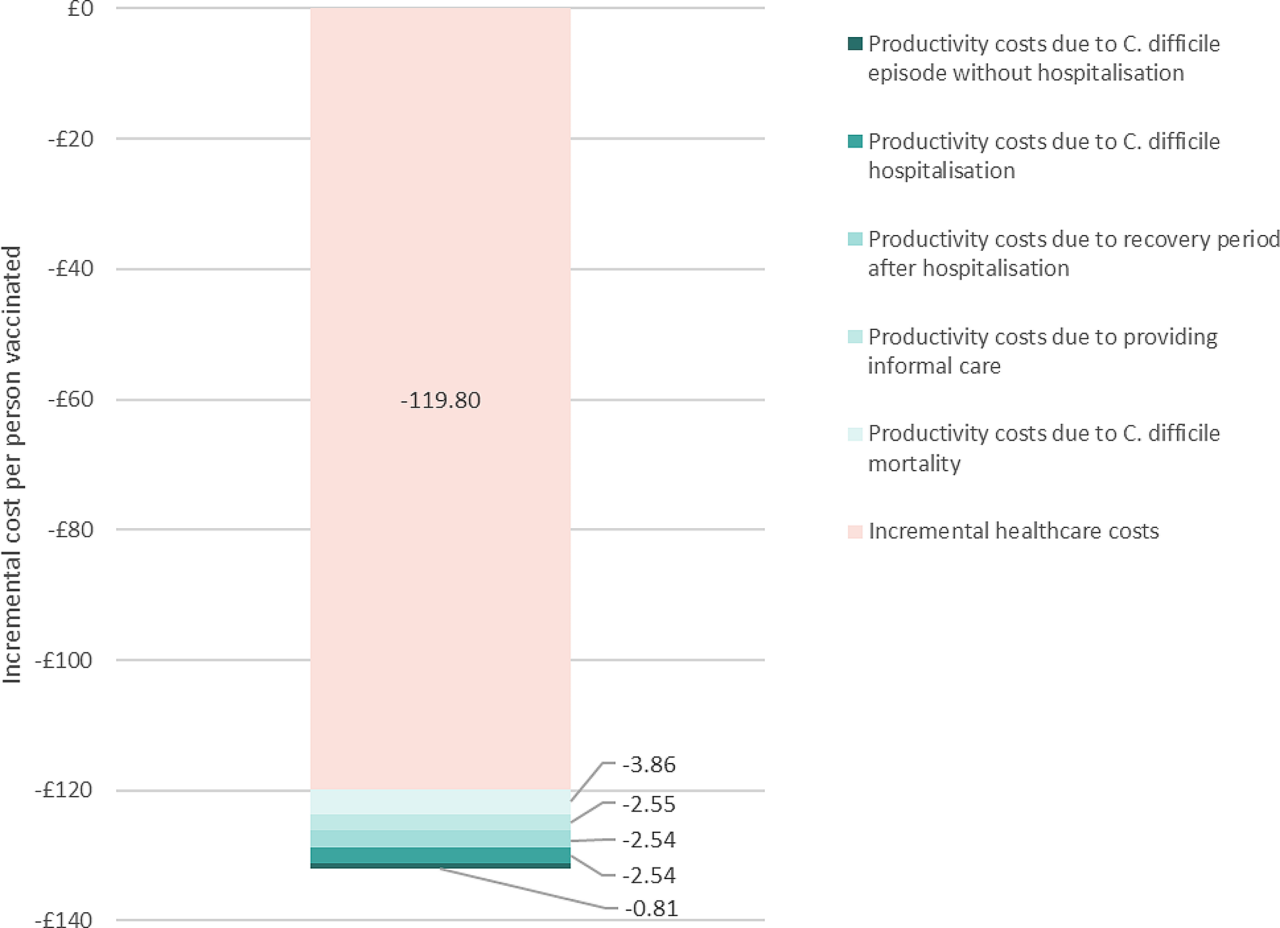
Difference in costs between standard of care and C. difficile vaccination strategy^*^, without and with productivity costs. * All results are net of the vaccine cost

The C. difficile model is an adapted version of a cost-effectiveness study by Lenoir-Wijnkoop et al. [17] of a probiotic for the prevention of C. difficile-associated diarrhoea in the UK. It uses a static decision-tree to describe the possible infection outcomes and associated costs among a hypothetical cohort of hospitalised adults ≥ 50 years receiving antibiotics (Fig. 1, Supplementary Material).

The model considers productivity costs due to (i) C. difficile episodes without hospitalisation, (ii) C. difficile episodes with hospitalisation, (iii) post-C. difficile hospitalisations recovery period, (iv) C. difficile-attributable mortality, causing loss in paid work for patients aged 50–64 and in unpaid voluntary work for patients aged 65+; (v) informal caregiving by family members to support a C. difficile patient. The time horizon of the analysis is one year.

### RSV model characteristics

**Fig. 2:**
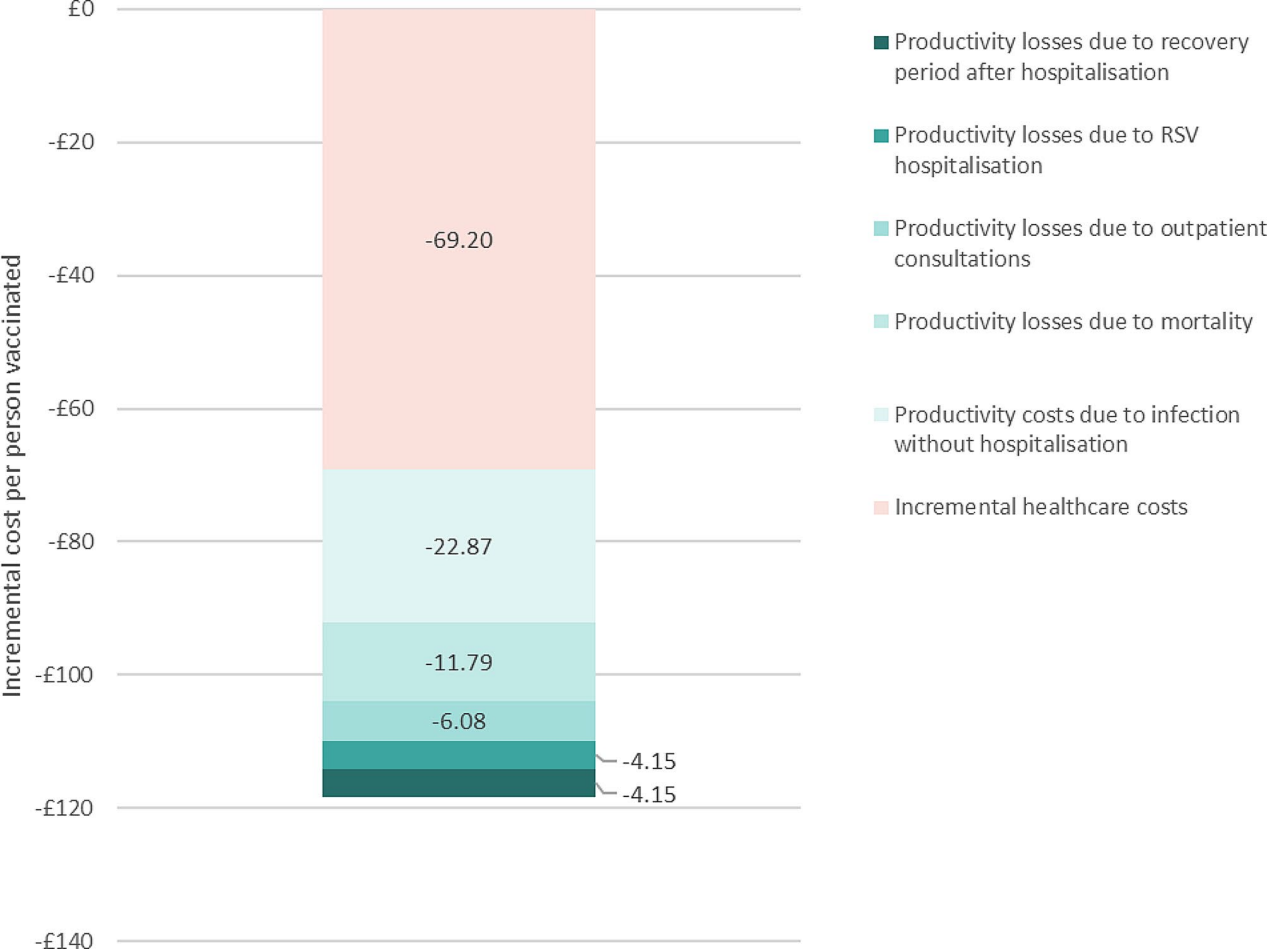
Difference in costs between standard of care and RSV vaccination strategy^*^, without and with productivity costs. * All results are net of the vaccine cost, which cancels out in the comparison

The RSV model is an adapted version of a cost-effectiveness analysis by Cromer et al. [18] of different immunisation strategies for RSV in children in England. It uses a static decision-tree comparing RSV outcomes and associated costs among a hypothetical cohort of children < 5 years of age (Fig. 2, Supplementary Material).

For RSV we included productivity costs due to losses in paid work by a family member (e.g. parent, guardian) to care for a child during (i) RSV-associated outpatient consultations, (ii) RSV infections without hospitalisation, (iii) RSV hospitalisations; (iv) post-RSV hospitalisation recovery period; and (v) patients’ productivity costs due to RSV-attributable mortality. The time horizon of the analysis is the patient’s lifetime.

### Model input data

Model inputs were based on a systematic review of economic evaluations of C. difficile and RSV interventions published between January 2000 and September 2021 (for search terms see Table 1, Supplementary Material), relevant statistics databases (e.g., Office for National Statistics, OECD Data) and official public health reports (e.g., Public Health England).

Where assumptions were needed in lieu of published data, these were verified for plausibility by three experts using a written questionnaire. Table 1 reports all input data used; additional details are provided in Supplementary Material 3.

**Table 1:** Population and disease epidemiology input data used in the models

Input parameter	Value (range)	Source
***C. difficile***		
Employment rate of UK population age 50 to 64 years, % employed for one hour or more per week	71 (57; 85)	Office for National Statistics 2021 [21]
*C. difficile* patients in working age of 50–64 years, %	17 (13;20)	Public Health England 2021 [14]
*C. difficile* patients aged 65 + years, %	72 (57; 86)	Public Health England 2021 [14]
Hypothetical vaccine efficacy, %	70 (50; 100)	Assumption based on expert opinion
Probability to develop *C. difficile*, %	2.3 (1.8; 2.7)	Calculation based on Lenoir-Wijnkoop 2014 [17]
Probability of a first recurrence, %	22 (18; 26)	Lenoir-Wijnkoop 2014 [17]
Probability of a second and third recurrence, %	35 (28; 42)	Lenoir-Wijnkoop 2014 [17]
Duration of *C. difficile* episode, days	10 (2;15)	Shen 2017 [22]; range based on expert opinion
*C. difficile* episodes in which patient is at home, %	20 (16; 24)	Assumption based on expert opinion
Duration of *C. difficile* hospitalization, days	7.8 (1.0; 60.0)	Bartsch 2012 [23], Steuten 2018 [24], Lenoir-Wijnkoop 2014 [17], Feuerstadt 2020 [25], Champredon 2020 [26]. Upper range based on expert opinion.
Duration of recovery period after *C. difficile* hospitalization, days	7.8 (1.0; 60.0)	Assumption same length as hospitalization
Probability to die from *C. difficile* until 30 days after each 30-day *C. difficile* episode, %	3.5 (2.8; 4.2)	Cronbach 2019 [27], Leal 2019 [28]
*C. difficile* patients requiring care during and/or after *C. difficile* episode, %	30 (20; 40)	Assumption, range based on expert opinion
Number of days *C. difficile* patients require care during and/or after *C. difficile* episode	7.8 (1; 15.6)	Assumption same length as hospitalization
Hours of informal care needed per day by *C. difficile* patients	3.6 (1.0; 7.2)	Based on proxy diseases. Asmus-Szepesi 2014 [29], Timonet-Andreu 2018 [30], Costa 2013 [31], Jowsey 2013 [32], Dunbar 2018 [33]
**RSV**		
Vaccine efficacy, %	70 (50; 100)	Cromer 2017 [18]
Employment rate of UK population age 16 to 64 years, % employed for one hour or more per week	76 (61; 92)	ONS 2020 [34]
Annual number of GP visits per year per child at risk (without vaccine), children aged below 5	1.2 (0.5; 0.19)	Cromer 2017 [18]
Number of working days lost due to GP consultation for RSV, children aged below 5	0.6 (0.3; 1.0)	Pouwels 2016 [35], Meijboom 2012 [36], Ginsberg 2008 [37]
Number of working days lost due to RSV infections without hospitalization, children aged below 5	3.3 (1.3; 14.0)	Acedo 2010 [38], Regnier 2013 [39], Ginsberg 2018 [37], upper range: CDC 2018 [40].
Average GP consultations per RSV episode, children aged below 5	1.5 (1.2; 1.8)	Assumption based on elicited expert opinion
Number of working days lost due to RSV hospitalization, children aged below 5	5.3 (1.0; 13.3)	Pouwels 2016 [35], Regnier 2013 [39], Acedo 2010 [38, 41], Lee 2018, Ginsberg 2018 [37], Leidy 2005 [42], McLaurin 2016 [43]. Upper range based on standard deviation reported by Leidy 2005.
Number of annual hospital admissions (without vaccination) per child at risk under 5 years	0.090 (0.007; 0.011)	Cromer 2017 [18]
Number of working days lost due to recovery period at home after hospitalization	5.3 (1.0; 10.6)	Ginsberg 2018 [37]. Upper range is twice the base case value.
Mortality rate 0–5 months (in-hospital deaths), %	0.20 (0.16; 0.02)	Shi 2017 [44]
Mortality rate 6–11 months (in-hospital deaths), %	0.09 (0.72; 1.08)	Shi 2017 [44]
Mortality rate 12–59 months (in-hospital deaths), %	0.07 (0.06; 0.08)	Shi 2017 [44]
**Cost Data**		
Productivity losses, per day	£163.0 (£130.24; 195.36)	OECD 2020 [45], ONS 2020 [46]; range based on percentage change.
Value of informal care work forgone of adults age 65+, per year	£3,326 (£2,661; £3,991)	Franklin and Hochlaf 2018 [47]; range based on percentage change.
Time of informal caregivers, per year, opportunity cost method	£20.35 (£16.28; £24.42)	OECD 2020 [45], ONS 2020 [46]; range based on percentage change.
Healthcare costs, *C. difficile* - Without vaccination program - With vaccination program^*^	£185 £165	Lenoir-Wijnkoop 2014 [17]
Healthcare costs, RSV - Without vaccination program - With vaccination program^*^	£99 £193	Cromer 2017 [18]

All costs are expressed for the 2021 cost year. ^*^Excluding vaccine cost

### Sensitivity analyses

We conducted deterministic sensitivity analyses to test the impact of lower and higher bound estimates of the input parameters on the incremental productivity cost estimates. Lower and higher bound values of the input parameters were derived from the literature, gathered from the experts’ responses to the questionnaire, or assumed to be +/-20% if no other sources were unavailable.

## Results

### C. Difficile

Estimates of the per person healthcare costs under the standard of care and the C. difficile vaccine strategy are £185.0 and £65.2, respectively. Productivity costs were estimated to amount to an additional £17.6 under the standard of care compared to £5.3 under the vaccination strategy (Fig. 3 Supplementary Material).

These results imply that, compared to the standard of care and net of the vaccine cost, the vaccine strategy reduces costs (i.e. generates savings) by £119.8 per person vaccinated when productivity costs are excluded, and by £132.1 when productivity costs are included (Fig. 1). The C. difficile vaccination strategy would therefore prevent an additional £12.3 in productivity costs per person vaccinated. The main driver of this value are the prevented productivity costs due to C. difficile mortality (£3.9 per person vaccinated).

### RSV

Estimates of the healthcare costs under the standard of care and the RSV vaccine strategy are £98.8 and £29.6 per person vaccinated, respectively. Productivity costs are estimated to amount to an additional £70.1 under the standard of care and £21.0 under the vaccination strategy (Fig. 4, Supplementary Material).

These results imply that, compared to the standard of care and net of the vaccine cost, the vaccine strategy reduces costs (i.e. generates savings) by £69.2 per person vaccinated when productivity costs are excluded, and by £118.2 when productivity costs are included (Fig. 2). The RSV vaccination strategy would therefore prevent an additional £49.0 in productivity costs per person vaccinated. The main driver of this value are the prevented productivity costs due to RSV infection episodes without hospitalisation (£22.9 per person vaccinated).

.

### Sensitivity analysis

The worst- and best-case value estimates of the incremental productivity costs of a C. difficile vaccination programme, compared to no vaccination, range between -£0.92 and -£190.7, respectively. In the case of an RSV vaccination programme, incremental productivity costs range between -£9.7 and -£281.3. Additional sensitivity analyses on individual productivity cost components are provided in the Supplementary Material 5.

## Discussion

This paper illustrates that productivity costs can have a substantial impact on the value assessment of vaccines against C. difficile and RSV infections. It also provides a granular insight into the main drivers of productivity value that would potentially be generated by each vaccination strategy compared to the standard of care in a UK setting. This understanding is critical to guide comprehensive evidence development of vaccination strategies which ultimately inform reimbursement decisions.

For a C. difficile vaccine strategy among hospitalised adults aged 50 and over, consideration of the incremental productivity costs per person vaccinated (-£12.3) increases the total expected cost savings by about 10% over and above the estimated incremental direct healthcare costs (-£119.3). For an RSV vaccine strategy targeting children below the age of 5, incremental productivity costs per person vaccinated (-£49) are expected to increase total cost savings by 76% compared to only considering incremental direct healthcare costs (-£69.2). Note that these results do not include vaccine costs in order to show the impact of incremental productivity costs over and above incremental health care costs before any potential value absorption in a vaccine price.

A particular strength of this work is that it considers a comprehensive set of short-term productivity costs during the acute infection phase as well as long-term productivity costs due to mortality. Furthermore, our analysis of productivity costs associated with a C. difficile vaccination strategy captures losses in unpaid work among patients in non-working age in addition to productivity losses in paid work. It shows losses in unpaid work represent 20% of the overall productivity costs saved by a C. difficile vaccination strategy (-£2.5 per person vaccinated).

Our analysis has some limitations. First, we did not pursue a de-novo health economic analysis because we intended to illustrate the potential relative impact of considering productivity costs in addition to healthcare costs. Therefore, we leveraged existing economic analyses and readily available data. Second, we did not include the vaccine cost for the reason mentioned above nor incremental health gains. These inputs would cancel out in our comparison (productivity costs versus no productivity costs), but as a consequence the results of this analysis should not be taken to reflect the cost-effectiveness of a particular vaccine. Third, for C. difficile, healthcare costs for age 50 + were not available so we used previously published costs for age 65+ [17]. This is likely to overestimate healthcare costs and underestimates productivity losses. Fourth, we chose to use the human capital approach and not a friction cost approach because (i) the duration of morbidity and recovery episodes for both diseases fall well within typical friction cost periods (95 ± 11 days for the UK [48]), (ii) mortality associated with each disease is limited yet for RSV pertains to children, making the human capital approach more relevant. Had we used the friction costs method then the productivity loss associated with C. difficile mortality would have been valued at £1.0 vs. £3.9 with the human capital approach. The sensitivity analyses also show that the incremental productivity costs of the vaccination programme can range quite substantially and further research to obtain more precise estimates will improve the ability to accurately reflect their inclusion in economic models. Finally, adult RSV vaccines were approved in the UK in 2023, but they were not at the time of conducting this analysis. Hence, this research is limited to infant RSV.

This paper contributes to the literature on the broader value of vaccination by showing that excluding productivity costs from a value assessment, such as a cost-effectiveness analysis, will fail to capture the full broader societal value of a vaccine. This is consistent with increasing academic consensus and international HTA guidelines on vaccines [49–54] that have argued for inclusion of broader value elements into vaccines’ evaluation. Of note, this same literature has also argued that consideration of societal benefits, where they are relevant, should be applied to all interventions funded by the same budget, to assure consistent decision making.

Finally, considering a growing pipeline of vaccines for older populations as well as the age of retirement going up in many high-income countries, including productivity losses from formal employment in adults including those aged 65 years or older is recommended to fully capture the life course value of vaccination.

## Conclusion

In a UK context, a C. difficile and an infant RSV vaccination programme, respectively, would prevent £12 and £49 in productivity costs for every person vaccinated. Given the potential magnitude of impact, it is recommended to further investigate and consider productivity costs in future cost-effectiveness analyses to assess this dimension of broader societal value and provide better-informed reimbursement decisions.

### Electronic Supplementary Material

Below is the link to the electronic supplementary material.

Supplementary Material 1

## Data Availability

All data used for the analysis are available from this paper and its supplemental material.
