# Supplementary material for: Impact of including productivity costs in economic analyses of vaccines for C. difficile infections and infant respiratory syncytial virus, in a UK setting
Source: Cost Eff Resour Alloc. 2024 Apr 30;22:34. doi: 10.1186/s12962-024-00533-4 (PMC11059668; doi:10.1186/s12962-024-00533-4)
Supplement: Supplementary file 1 — Supplementary Material 1 [file 12962_2024_533_MOESM1_ESM.docx]

**SUPPLEMENTARY MATERIAL**

**S1. C. difficile and RSV model characteristics**

In the C. difficile model, hypothetical patients are either vaccinated or unvaccinated against C. difficile. Patients who acquire C. difficile can either die, fully recover, or have a C. difficile recurrence. The model allows for a maximum of three recurrences each of occurring during three different 8-week periods (49). After each recurrence, there is a probability that the patient dies due to C. difficile, recovers from C. difficile, or has another recurrence. After the third recurrence, patients in the model either die or fully recover. No differentiation in disease severity between C. difficile episodes is made. Hospitalised patients who do not acquire C. difficile have 0% probability of C. difficile-attributable death and do not incur healthcare costs, productivity losses, or QALY decrements associated with C. difficile. The time horizon of the analysis is one year.

In the RSV model, hypothetical patients are either vaccinated or unvaccinated against RSV. Children who develop RSV have a probability to require in-patient hospital treated or by treated outside of the hospital setting. Non-hospitalised children with RSV are assumed to recover; hospitalised children either recover or may die from RSV. Children who do not develop RSV are not at risk for RSV-attributable death and they and/or their caregivers do not incur healthcare costs or productivity losses. A lifelong time horizon was used starting from three months of age.

**S2. Search strategy**

**Supplementary Table 1:** Key words / search terms used in the systematic literature search

| **Search #** | **Search engine** | **Query** |
| --- | --- | --- |
| 1 | PubMed | (respiratory syncytial virus [Title/Abstract] OR paramyxovirus [Title/Abstract] OR respiratory tract infections [Title/Abstract] OR RSV [Title/Abstract] OR bronchiolitis [Title/Abstract]) AND (Economic evaluation [Title/Abstract] OR Economic model [Title/Abstract] OR Health Technology Assessment [Title/Abstract] OR HTA [Title/Abstract] OR Incremental cost effectiveness ratio [Title/Abstract] OR ICER [Title/Abstract] OR Cost effectiveness [Title/Abstract] OR Cost benefit [Title/Abstract] OR Cost consequence [Title/Abstract] OR Cost minimization [Title/Abstract] OR Cost per QALY [Title/Abstract] OR Cost utility [Title/Abstract]) |
| 2 | PubMed | (clostridium difficile [Title/Abstract] OR c difficile [Title/Abstract] OR c diff [Title/Abstract] or cdf [Title/Abstract]) AND (Economic evaluation [Title/Abstract] OR Economic model [Title/Abstract] OR Health Technology Assessment [Title/Abstract] OR HTA [Title/Abstract] OR Incremental cost effectiveness ratio [Title/Abstract] OR ICER [Title/Abstract] OR Cost effectiveness [Title/Abstract] OR Cost benefit [Title/Abstract] OR Cost consequence [Title/Abstract] OR Cost minimization [Title/Abstract] OR Cost per QALY [Title/Abstract] OR Cost utility [Title/Abstract]) |
| 3 | Google Scholar | allintitle: respiratory syncytial virus "Economic evaluation" OR "economic model" OR "Health Technology Assessment" OR HTA OR "Incremental cost effectiveness ratio" OR ICER OR "Cost effectiveness" OR "cost benefit" OR "cost consequence" |
| 4 | Google Scholar | allintitle: respiratory syncytial virus "cost minimisation" OR "cost per QALY" OR "cost utility" |
| 5 | Google Scholar | allintitle: RSV "Economic evaluation" OR "economic model" OR "Health Technology Assessment" OR HTA OR "Incremental cost effectiveness ratio" OR ICER OR "Cost effectiveness" OR "cost benefit" OR "cost consequence" |
| 6 | Google Scholar | allintitle: RSV "cost minimisation" OR "cost per QALY" OR "cost utility" |
| 7 | Google Scholar | allintitle: clostridium difficile "Economic evaluation" OR "economic model" OR "Health Technology Assessment" OR HTA OR "Incremental cost effectiveness ratio" OR ICER OR "Cost effectiveness" OR "cost benefit" OR "cost consequence" |
| 8 | Google Scholar | allintitle: clostridium difficile "cost minimisation" OR "cost per QALY" OR "cost utility" |
| 9 | Google Scholar | allintitle: c difficile "Economic evaluation" OR "economic model" OR "Health Technology Assessment" OR HTA OR "Incremental cost effectiveness ratio" OR ICER OR "Cost effectiveness" OR "cost benefit" OR "cost consequence" |
| 10 | Google Scholar | allintitle: c difficile "cost minimisation" OR "cost per QALY" OR "cost utility" |

**S3. Input data**The probability among hospitalised adults who take antibiotics to develop C. difficile was 2.3% (13). A C. difficile episode in our model lasted 10 days (18) and a hospitalisation for C. difficile 7.8 days (13, 19–22). The probability to die of C. difficile within 30-days was 3.5% after each episode (23, 24).

For RSV, children had an annual average number of GP consultations of 0.12 per child (14). The number of symptomatic RSV infections was calculated using the assumption that per RSV infection, an average of 1.5 GP consultations take place, leading to 0.08 infections per child per year. An RSV infection without hospitalisation lasted 3.29 days (33–35) and a hospitalisation 5.31 days (31, 33–35, 37–39). The in-hospital mortality rate ranged from 0.2% for 0–5 months old to 0.7% for 12–48 months old (40).

As the vaccines for C. difficile and RSV are not yet available, we assumed a hypothetical vaccine efficacy of 70% (14). Employment rates specific for the age groups (age 50 + for C. difficile, all ages for RSV) were identified from the Office for National Statistics (17, 30). For C. difficile, the share of patients in specific age groups to whom productivity losses apply were taken from a report by Public Health England (2021) (10).

Costs included are the productivity losses per day, the hourly value of informal care provided (for C. difficile), the value of informal care lost, and lifetime lost income (for RSV). The healthcare cost to which the productivity losses were added to arrive at the societal costs were based on Lenoir-Wijnkoop et al. (2014) (13) for C. difficile, and on Cromer et al. (2017) (14) for RSV.
Cromer et al. reported the health care costs in the usual care group, the maximum cost-effective price for the vaccine, and the willingness-to-pay [19]. Based on these data, the vaccine efficacy, and input data provided, we calculated the approximate health care costs for the vaccine group using the ICER equation.

**S4. Supplementary figures**

**Supplementary Fig. 1:** C. difficile decision-tree model structure (+) the arms from above starting from “vaccine” are repeated.


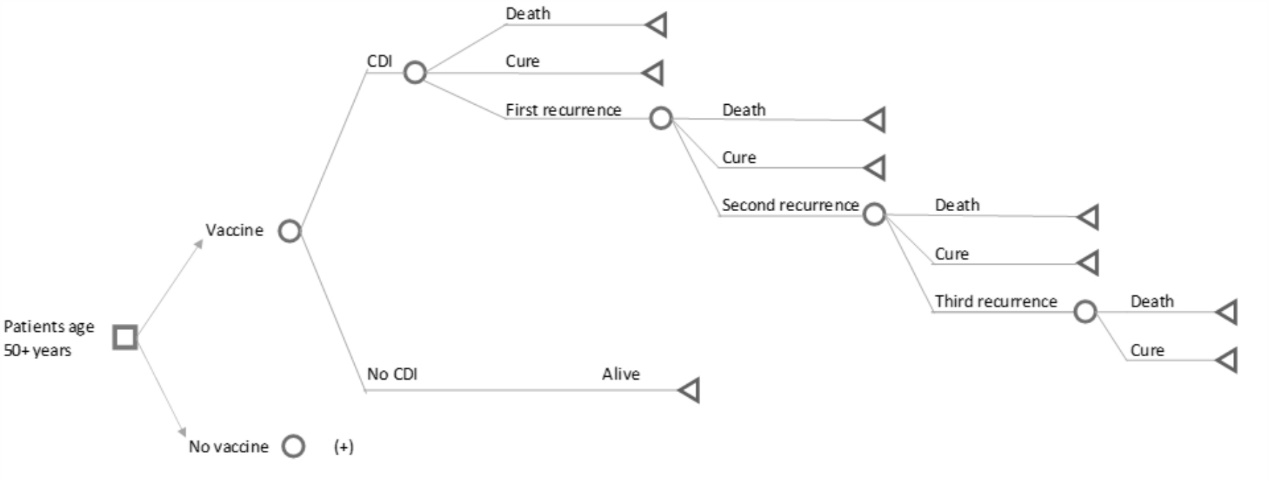


**Supplementary Fig. 2:** Decision-tree for RSV used for analysing productivity losses (+) the arms from above starting from “vaccine” are repeated. Survival with sequelae was analysed as a scenario.


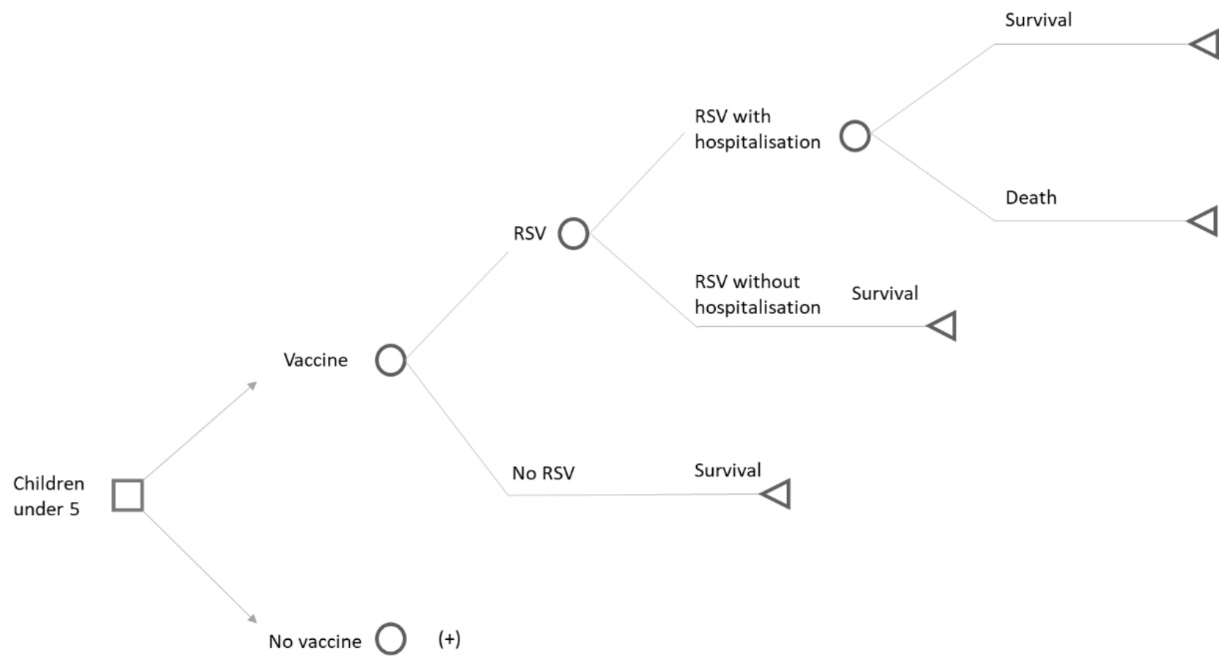


**Supplementary Fig. 3:** Direct healthcare costs and productivity costs under the standard of care and C. difficile vaccination strategy


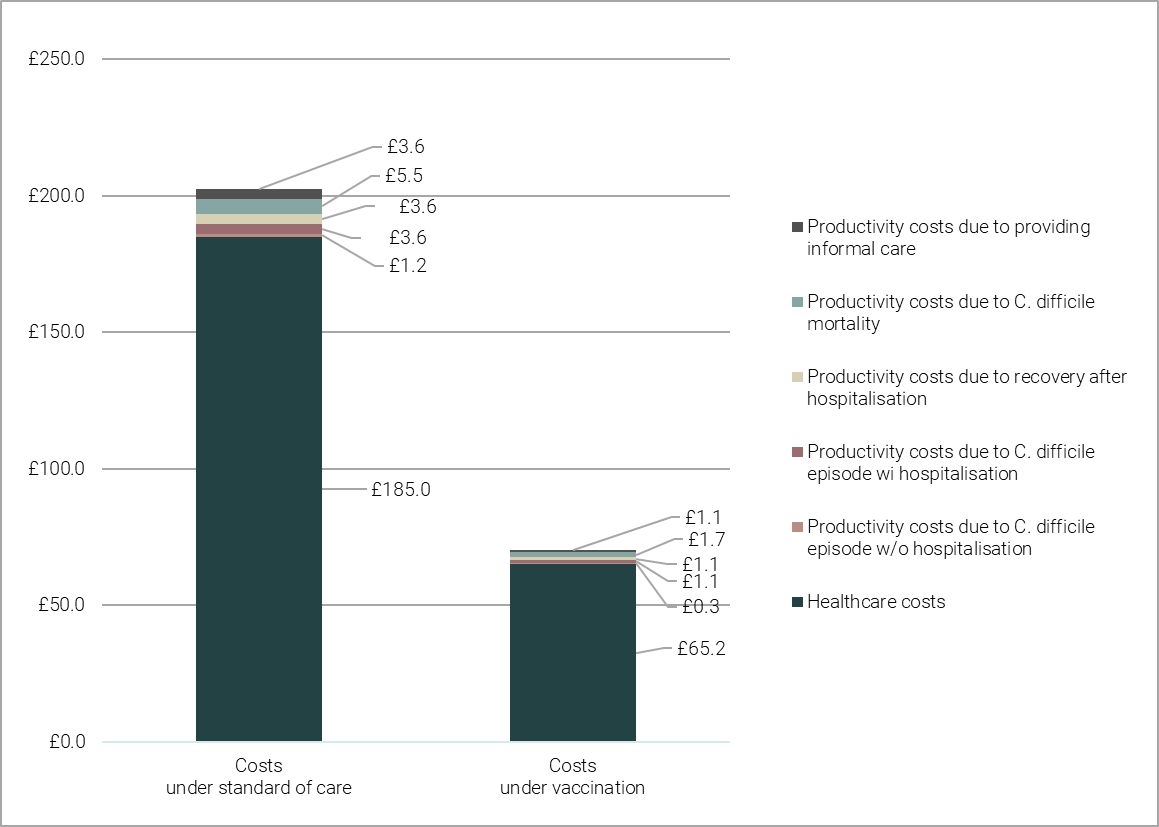


**Supplementary Fig. 4:** Direct healthcare costs and productivity costs under the standard of care and C. difficile vaccination strategy


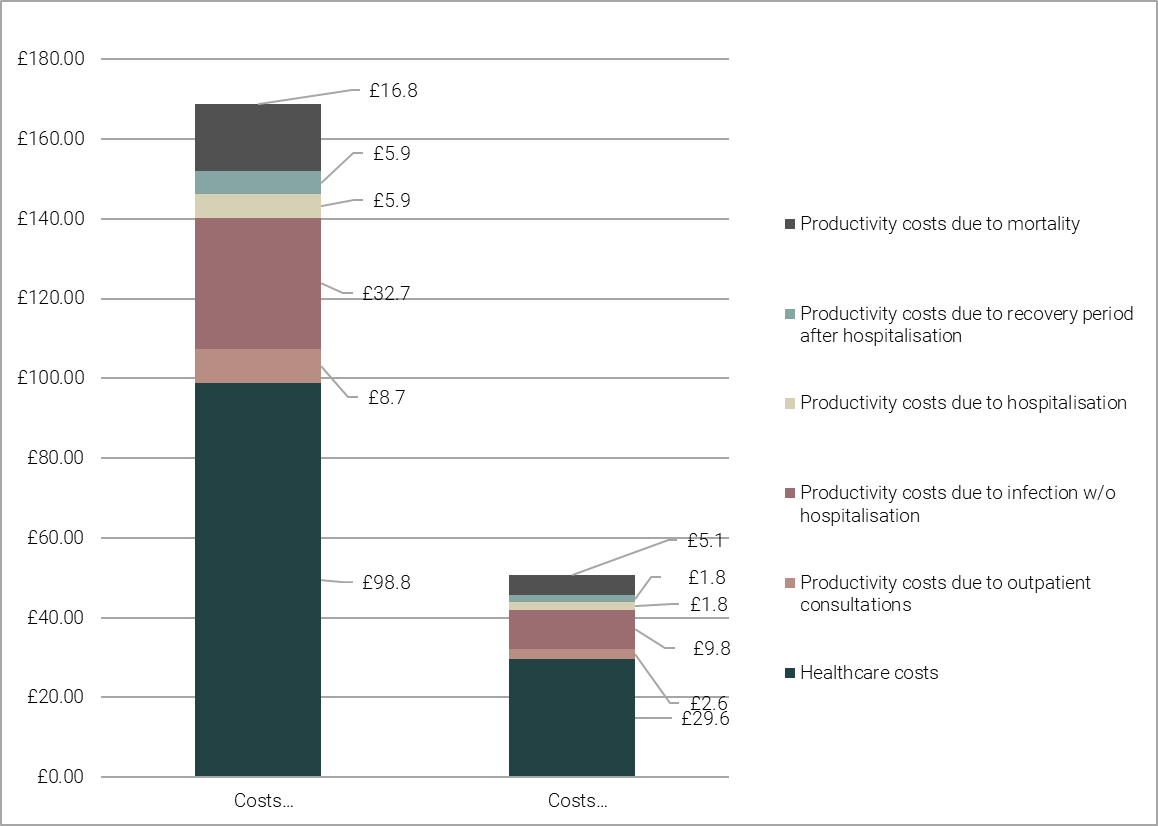


**S5. Sensitivity analysis**

Supplementary Table 2 shows the ranges of uncertainty around the baseline incremental productivity costs estimates (i.e. difference in productivity costs between standard of care and each vaccination strategy).

| **Results sensitivity analysis** | Baseline | Worst-case estimates | Best-case estimates |
| --- | --- | --- | --- |
| **Incremental productivity costs C. difficile vaccination programme** | **-£12.3** | **-£0.92** | **-£190.7** |
| Incremental productivity costs due to *C. difficile* episode without hospitalisation | -£0.8 | -£0.03 | -£5.2 |
| Incremental productivity costs due to C. difficile episode with hospitalisation | -£2.5 | -£0.08 | -£65.9 |
| Incremental productivity costs due to recovery after hospitalisation | -£2.5 | -£0.08 | -£65.9 |
| Incremental productivity costs due to *C. difficile* mortality | -£3.9 | -£0.7 | -£17.4 |
| Incremental productivity costs due to providing informal care | -£2.5 | -£0.02 | -£36.2 |
| **Incremental productivity costs RSV vaccination programme** | **-£49.0** | **-£9.7** | **-£281.3** |
| Incremental productivity costs due to RSV episode without hospitalisation | -£22.9 | -£5.2 | -£166.8 |
| Incremental productivity costs due to RSV episode with hospitalisation | -£4.2 | -£0.3 | -£25.7 |
| Incremental productivity costs due to recovery period after hospitalisation | -£4.2 | -£0.3 | -£20.5 |
| Incremental productivity costs due to RSV mortality | -£11.8 | -£3.4 | -£34.9 |
| Productivity costs due to outpatient consultations | -£6.1 | -£0.5 | -£33.3 |
